# Expression of Concern: Age Related Changes in NAD+ Metabolism Oxidative Stress and Sirt1 Activity in Wistar Rats

**DOI:** 10.1371/journal.pone.0263555

**Published:** 2022-01-31

**Authors:** The PLOS ONE Editors

After this article [1] was published, concerns were raised about western blots in Figs 2, 5, 7 and 8, microscopy images in Figs 5 and 7, and charts in Fig 1.

Specifically:

- In Figs 2A-D, 5Bi-iv, 7Bi-iv, and 8A-D in this article, a number of blots and/or bands appear similar to each other across two or more figure panels, in some cases with 180° rotation. Some blots and/or bands in these figures of [1] also appear similar to images in Fig 2 in [2], and Fig 2a in [3].
- Several panels in Figs 5C and 7C appear similar, in some cases with 180° rotation.
- In Fig 1A and 1B, the error bars for different organs appear similar within each age group.

In response to queries about these figures, the first author has indicated they stand by the validity of the results reported in the article. PLOS has not yet received conclusive information about the availability of the underlying data for the experiments in question.

Follow up on these issues is ongoing; in the meantime, the *PLOS ONE* Editors issue this Expression of Concern.

## References

[1] Braidy N, Guillemin GJ, Mansour H, Chang-Ling T, Poljak A, Grant R (2011) Age Related Changes in NAD+ Metabolism Oxidative Stress and Sirt1 Activity in Wistar Rats PLoS ONE 6(4): e19194. doi: 10.1371/journal.pone.0019194 21541336 PMC3082551

[2] Braidy N., Poljak A., Grant R. et al. Mapping NAD+ metabolism in the brain of ageing Wistar rats: potential targets for influencing brain senescence. Biogerontology 15, 177–198 (2014). doi: 10.1007/s10522-013-9489-5 24337988

[3] Braidy N, Poljak A, Grant R, Jayasena T, Mansour H, Chan-Ling T, Smythe G, Sachdev P and Guillemin GJ (2015) Differential expression of sirtuins in the aging rat brain. Front. Cell. Neurosci. 9:167. doi: 10.3389/fncel.2015.00167 26005404 PMC4424846

